# Polyethylene Nail Brace for Ingrown Toenails Treatment: A Randomized Clinical Trial

**DOI:** 10.3390/ijerph17217741

**Published:** 2020-10-23

**Authors:** Salvador Márquez-Reina, Inmaculada Palomo-Toucedo, María Reina-Bueno, José Manuel Castillo-López, Javier Ramos Ortega, César Calvo-Lobo, Daniel López-López, Gabriel Domínguez-Maldonado

**Affiliations:** 1 Nuestra Señora de la Oliva Foot Clinic, Bda. Ntra. Sra. de la Oliva Bq. 24, Bajo B, 41013 Sevilla, Spain; podosalva@hotmail.com; 2 Departamento de Podologia, Universidad de Sevilla, Calle Avicena, s/n, 41009 Sevilla, Spain; ipalomo@us.es (I.P.-T.); mreina1@us.es (M.R.-B.); jmcastillo@us.es (J.M.C.-L.); jrortega@us.es (J.R.O.); gdominguez@us.es (G.D.-M.); 3 School of Nursing, Physiotherapy and Podiatry, Universidad Complutense de Madrid, 28040 Madrid, Spain; cescalvo@ucm.es; 4 Research, Health and Podiatry Group, Department of Health Sciences, Faculty of Nursing and Podiatry, Universidade da Coruña, 15403 Ferrol, Spain

**Keywords:** onychocryptosis, nail curves, nail brace, orthonyxia, spicule

## Abstract

**Background:** Onychocryptosis is one of the most prevailing onychopathies and one of the usual reasons for visiting podiatry clinics. In this research, we aim to evaluate the effectiveness of a procedure of nail reeducation technique via a strip of polyethylene in subjects with stage I or IIa onychocryptosis, in which pathological toenail curves are present. **Methods:** This research was a randomized clinical trial (ACTRN12615000834550). The sample was made up of 94 cases of stage I or IIa onychocryptosis, according to the Mozena classification. Briefly, 46 cases were treated with the combination of a spicule technique and nail brace with a polyethylene plastic strip, and 48 were only treated with the spicule technique. **Results:** The combination of the spicule technique and the nail brace technique with a strip of polyethylene had a significantly lower recurrence rate compared to that achieved with just the spicule technique, twelve months after the beginning of the study (N.S. = 0.000 for α = 0.05). The change in the nail width achieved with the nail brace technique, twelve months after the beginning of the study, was statistically significant (N.S. = 0.000 for α = 0.05). **Conclusions:** The recurrence rate of the spicule technique alone was significantly higher than the combined technique of spicule with nail brace. A nail brace with a strip of polyethylene reduces the recurrence rate of onychocryptosis.

## 1. Introduction

Of all the onychopathies described, onychocryptosis is undoubtedly one of the most prevailing conditions and one of the usual reasons for visits to podiatry clinics. It was defined for the first time in 1845 as “a nail which grows towards the inside of the flesh” as a consequence of an excessive curvature, which causes pain and inflammation of the surrounding tissues. It has a prevalence of 20% of the patients who visit for a foot pathology [1]. In a study carried out with 10,900 podiatry patients, it was indicated that 61% of them (6754) presented symptomatic nail conditions. There is a predilection for the first toe of the foot and its peroneal edge (with a proportion of 2–5:1) [2], although it can affect any toe and either of the two edges [3,4].

In 2002, Mozena [5] classified this pathology into four stages: stage I or inflammatory, stage II or abscess, which is divided into two substages, stage IIa and stage IIb, and stage III. In the initial stages of the onychocryptosis (stages I and IIa), conservative treatments are efficient in reducing painful processes, solving a great number of cases, which is why they are considered first-choice treatments [6].

Nail reeducation techniques can be varied: applying cotton impregnated with alcohol below the nail edges, plugging with gauze, applying plastic cannulas, applying dental floss, acrylic resin nails, metallic nail braces, or plastic nail braces.

In this study, we investigate the effectiveness of a procedure of nail reeducation in subjects with onychocryptosis at stages I or IIa, who present pathological nail curves, using a nail brace technique carried out with a strip of polyethylene (polyethylene orthonyxia) stuck to the nail with cyanoacrylate.

## 2. Materials and Methods

### 2.1. Design and Sample

We have carried out an ongoing, phase IV, blinded parallel-group, randomized clinical trial with an endpoint classification of efficacy study 1:1 allocation ratio conducted at an only-podiatry medical center. No changes were made in the proposed methodology because the original protocol was not altered. Furthermore, the Consolidated Statement for Reporting Trials (CONSORT) statement and checklist were considered. The study factor was the type of treatment, which had two values: one of which was the combination of the spicule technique and the nail brace technique with a strip of polyethylene (experimental group), and the other just the spicule technique (control group). The assignation of each subject to the randomized clinical trial was computer-generated by the statistic software Epidat version 4.2 (Consellería de Sanidade, Xunta de Galicia, España; Organización Panamericana de la salud (OPS-OMS); Universidad CES, Colombia).

### 2.2. Subjects

The sample was made up of 94 cases of mild pincer toenail onychocryptosis at stages I and IIa, according to Mozena’s classification [5]. Sixty-four people took part: 17 men and 47 women. Forty-six cases made up the experimental group and 48 cases the control group. The mean age was 54.9 years old for the experimental group and 57.96 years old for the control group.

### 2.3. Ethics Considerations

This randomized clinical trial was approved by the local Experimental and Ethics Committee of the University of Seville, in Seville, Spain, with 20 February 2012 as the date of signing, and it was recorded in the Australian New Zealand Clinical Trials Registry (ACTRN12615000834550). All voluntary patients provided their written consent form before the randomized clinical trial started. Human research ethical standards, according to the Helsinki Declaration, the human rights and biomedicine statements regarding the Convention of the Council of Europe, as well as the human genome and rights considered by the UNESCO Universal Declaration and other appropriate national or institutional organizations, were respected.

### 2.4. Sample Size

G*Power version 3.1.9.7 statistics power tool software was used to analyze and establish the sample size; a 90% confidence level, with 0.95 power and a 0.05 alpha level, was utilized. Additionally, a 15% sample loss was also adivsed. This procedure was stipulated based on the experience of previous studies carried out by the authors. Finally, the necessary sample size obtained was thirty cases each for the control and experimental groups, making a total of sixty cases, although more cases were recruited due to the estimated magnitude of data loss. One hundred cases were randomly separated into two groups of fifty cases, in which six cases were discarded from the study due to experimental death; of these, four were from the study group and two from the control group.

### 2.5. Procedures

Having obtained the patients’ informed consent, which fulfilled the inclusion criteria, a complete clinical history was carried out. Next, a straight nail cut was done at the hyponychium level. Later, the width of the nail was measured. Nail hyponychium was taken as a reference to affect the measurement of the nail width, as it is a fixed point of reference in all people. A caliber was used for the measurement. Later, the spicule technique of the nail edge or edges affected by onychocryptosis was carried out. The nail portion that injures the periungual soft tissues, causing onychocryptosis, is called a spicule.

We now describe the technique of cutting the nail spicule [7,8]. After disinfecting the area, the edge of the nail is milled in order to weaken the sheet and facilitate cutting. A first oblique cut is made with a nail clipper and finished with a number 15 scalpel, pointing dorsally. The spicule is cut to the back of the affected area and extracted with the help of fine forceps, ensuring that the end of the cut does not form an angle that could create a harpoon.

This treatment was performed at least once in all the subjects belonging to the study, both in the study group and in the control group. Local cures with povidone–iodine were indicated once a day for a week.

In the experimental group, in addition to the previous treatment, a nail brace was applied to correct the nail layer. The polyethylene nail brace consisted of applying a flat strip of this type of plastic between the free edge and the middle area of the nail, which, being stuck on the nail with cyanoacrylate and having elastic memory toward the horizontality, tends to modify its curvature. The principles of action are based on the tensile forces exercised by the plastic strips, which act directly on the proximity of the nail matrix and will accompany the growth of the nail, affecting traction in its more distal zones.

The nail brace technique, with a plastic strip, is bloodless, painless, and enables carrying out any kind of movement and using any type of footwear; it is easy to apply and of little economic cost [9].

**Figure 1:**
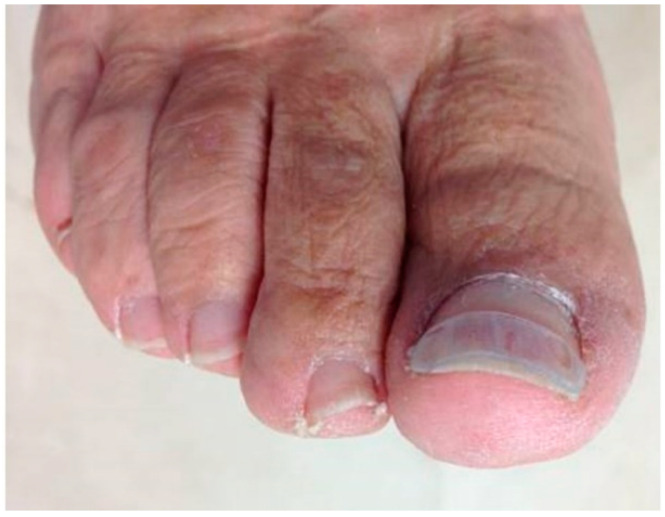
Strip of polyethylene treatment in ingrown toenails.

The strips of polyethylene were obtained from 0.5 mm thick polyethylene sheets. It was necessary to measure the width of the nail strip with a tape measure to adjust the size of the nail brace to the needs of each case (Figure 1). The length of the polyethylene strip was 2 mm less than the length of the nail from one side to the other, 1 mm less for each end. The width with which the plastic strip was cut was a third of the measure of the nail from the eponychium to the free edge.

**Figure 2:**
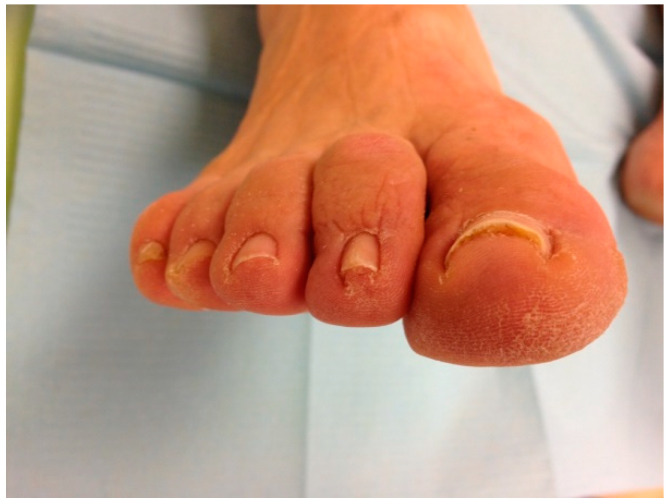
Initial appearance of the 1st toenail.

**Figure 3:**
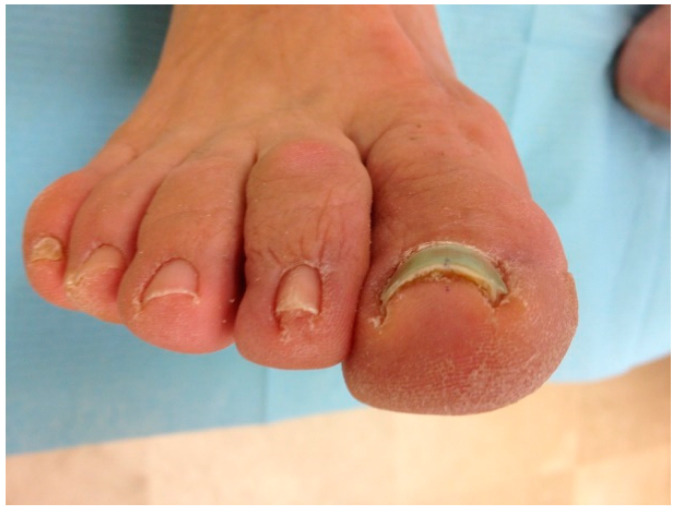
Appearance at 6 months.

**Figure 4:**
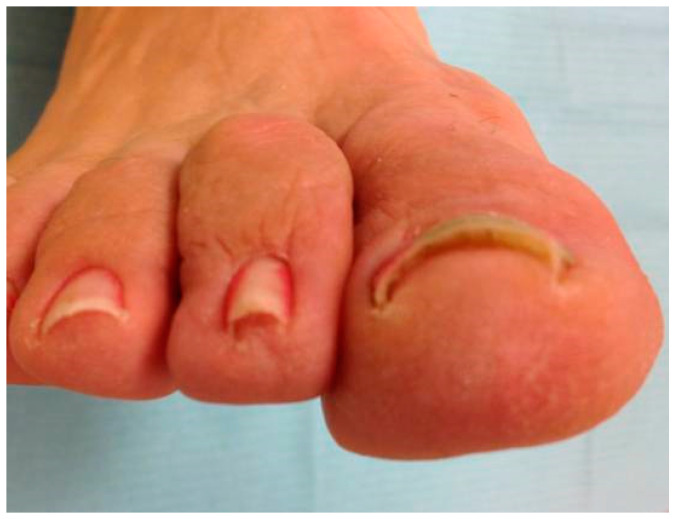
Appearance at 12 months.

The growth time of the toenails from the matrix to the free edge can be 12–18 months, depending on the location. The modification of the curvature of the nail from the eponychium to the free edge was observed, measuring the modification of the nail curvature at the level of the hyponychium. We have followed the same treatment time as a similar investigation of nail reeducation with plastic sheet orthonyxia, which was 6 months [9]. All the subjects who made up the experimental group were given a minimum of three nail braces. The first when starting the study, then another after two months, and, lastly, the third after four months. In the event of an accidental detachment, a new strip of polyethylene was put into place, and, in this case, the subject undertook to notify us when this happened. We considered the follow-up period to start once the treatment period had ended since the treatment was carried out over 6 months. The follow-up period was another 6 months. This follow-up period is also that of diverse authors consulted in the bibliography [10,11,12,13,14,15]. All the study subjects were given a check-up appointment every 2 months during a year. At these check-ups, the evolution was confirmed, and the nail width was measured in the study group’s subjects after 2, 4, 6, and 12 months (Figure 2, Figure 3 and Figure 4).

At these check-ups, the subjects who made up the control group only had the nail cut done at the level of the hyponychium, without spicule treatment, if they did not present recurrences.

### 2.6. Data Collection and Analysis

The data were analyzed with the SPSS^®^ computer pack, version 17.0 for Windows^®^ (SPSS Science, Chicago, IL, USA).

The Kolmogorov–Smirnov test was used to determine the normality of certain variables. The Mann–Whitney U-test and the Wilcoxon *t*-test were used.

The Pearson correlation test was employed for the study of measurement reliability.

Its reliability has been assessed, repeating the measurement process with the aim of analyzing the concordance between the different measures. We studied different aspects of reliability: intraobserver concordance and interobserver concordance.

## 3. Results

Table 1 shows the recurrence values during the period of treatment and follow-up, respectively, in the two study groups.

During the treatment period, in the experimental group, there were only 13 cases with some recurrences. On the other hand, in the control group, almost all subjects had at least one recurrence. Only 10 cases of the experimental group had any recurrence during the follow-up period (Table 1).

Taking into account the follow-up period after treatment, in the group with the nail brace, it was noted that 56.52% of the sample did not repeat any symptomology during that year. On the other hand, with only the spicule treatment, 95.83% had to revisit the podiatrist for treatment. This meant that not applying the nail brace after spicule treatment led to 11.22 times more recurrences in the control group than in the study group, beyond even the phase of treatment, taking measures throughout all of a year.

The Mann–Whitney U-test showed significance values of less than 0.001 when compared to the number of recurrences in both groups, both considering and not considering the follow-up period.

With respect to the measuring of the nail width, Table 2 shows the values of nail widths recorded in the study group during the treatment and the follow-up. It can be noted, descriptively, that there was an increase in this width during the period of treatment.

The measurements of nail width in all the assessments carried out during the application of the treatment (up to 6 months), as well as the comparison of the nail width 6 months after finishing the treatment (follow-up period), showed significant differences with respect to the initial width (<0.001) using the Wilcoxon test. A progressive increase can be noted in the nail width during the treatment period, which is related to a change in the nail’s morphology, reducing its curve. However, 6 months after applying the treatment, this value decreases. That is to say, the nail layer shows a tendency to partially recover its initial curve, showing values very similar to those achieved after 2 months of treatment. However, despite this, the percentage of recurrence of pain symptoms did not increase at the end of the follow-up period. We plan to extend the follow-up period in future studies to be able to check if the tendency of the nail over time is to equal the initial width and if there are recurrences of the symptoms.

For the study of the reliability of the measurements, we carried out five measurements of 10 different nails at three different moments—the first measurement after a week and then after two weeks—in such a way that we could verify an index of intrasubject reliability. Likewise, a second examiner did the same measurements to value intersubject reliability. We studied the Pearson correlation index, which showed values of between 0.99 and 1 for all the measurements studied, both for the intraobserver measurements and the interobserver measurements. This guaranteed the reliability of the measurement technique used.

## 4. Discussion

Among the studies that have carried out on nail reeducation techniques with a plastic strip, according to Effendy et al. [16], the effectiveness rate obtained was 100% during a follow-up that went from 3 to 6 months. However, it has to be taken into account that this was a study done with a sample of 3 cases, and the application time of the treatment was not specified. On the other hand, Di Chiacchio et al. [9], with a sample of 25 cases and 6 months of treatment, established an effectiveness rate of 100% during a follow-up period that went from 3 to 6 months. In both studies, the follow-up period was not exactly the same as in this study as they indicated that it went from 3 to 6 months, while in this study, it was 6 months in all cases. Additionally, our sample size differed quite a lot from the two previous studies. Nor was the stage of the onychocryptosis of the subjects included in the sample specified.

Studies that have evaluated the effectiveness of other nail brace techniques and nail reeducation techniques are referenced to be able to make comparisons with this study (Table 3).

Most of these studies did not have a research method similar to this study. Many had a fundamentally descriptive aim, showing the results obtained with a specific technique or with various techniques at the same time in the same study; a study group that was compared with a control group was lacking. It was thus difficult to interpret their results. Other publications that were found were based purely on empirical data and, therefore, did not offer scientific data on the results or the recurrence rates.

As to the effectiveness rate of the spicule technique alone, few studies have been published (Table 4). When analyzing the nail width variable, it has been noted that an average difference of 1.8798 mm was obtained after 6 months and 0.8185 mm after 12 months. We compared the nail width measurements of the subjects of the study group after 6 months and 12 months and we noted descriptively that there was a decrease of this width, the average decrease being −1.0613 mm. This is the period in which the subjects did not have the nail brace in place. That is to say, it seems that the nails treated have a certain capacity of memory and tend to curve slightly with the passing of time.

Within the studies that have analyzed changes in the nail width (Table 4), in the study carried out by Di Chiacchio et al. [9], they found an average difference of a change in nail width of 3.04 mm at the end of the 6-month treatment period. This was the same time carried out in this study, although they used a greater number of applications of orthonyxia in each subject, specifically six, one for each month treated. In this clinical trial, we applied a nail brace every two months. That is to say, a total of three orthonyxia unless there was an accidental detachment, in which case a new nail brace was put into place. They were also nail braces of different plastics, using the so-called “Sistema clip” of Luga Suministros Médicos S.L. They did not refer to the average nail width that the subjects had at the end of the follow-up period, data that we did record in this study, which is why it has not been possible to make comparisons. Nor did they have a control group.

As was indicated in the study of Di Chiacchio et al. [9], in this clinical trial, we have noted that the greatest average of correction of nail curves took place in the first 2 months of treatment; that is, the average nail correction was significantly higher in the interval of time between the beginning of the study and after 2 months.

Finally, there are some limitations in this clinical trial that have to be mentioned; these are related to (1) restricted sample size, (2) brief follow-up duration, and (3) not being a multicenter study.

## 5. Conclusions

The recurrence rate of the spicule technique alone is significantly higher than the combined technique of spicule with a nail brace. A nail brace with a strip of polyethylene reduces the recurrence rate of onychocryptosis.

## Figures and Tables

**Table 1:** Recurrence data of ingrown toenails in both groups.

	Treatment Period		Follow-Up Period	
	No Recurrence	Recurrence	No Recurrence	Recurrence
Experimental Group	33 (71.74%)	13 (28.26%)	36 (78.26%)	10 (21.74%)
Control Group	2 (4.17%)	46 (95.83%)	4 (8.33%)	44 (91.67%)

**Table 2:** Descriptive parameters of the nail width measurements in the experimental group.

	Initial Nail Width	Nail Width after 2 Months	Nail Width after 4 Months	Nail Width after 6 Months	Nail Width after 12 Months
Mean	12.86 ± 2.70	13.88 ± 2.55	14.41 ± 2.57	15.01 ± 2.67	14.03 ± 2.57
Minimum	4.83	6.20	6.88	7.04	5.49
Maximum	16.78	17.60	17.98	18.25	17.52

All measurements are in millimeters.

**Table 3:** Studies of the effectiveness of other nail brace techniques and nail reeducation techniques.

Nail Brace Techniques					
Studies	Year	Nº Cases	Treatment Time	Follow-Up Time	Effectiveness Rate
Van Oirschot et al. [17]	1994	85	Average 9 sessions	-	76.5% *
Kim and Sim [13]	2003	14	1 month	12 months	100%
Harrer et al. [11]	2005	21	-	6–12 months	81% *
Erdogan [18]	2008	7	-	6 months	100%
Iribarren and Delgado [19]	2006	10	-	-	100%
Cabo and Macián [20]	2007	-	141 days	-	82% *
Erdogan and Erdogan [21]	2006	21	4.1 ± 2.36 months	2 years	71.4% *
Kruijff et al. [10]	2008	47	-	12 months	82.98% *
Ishibashi et al. [22]	2008	14	-	3 months	100%
Kim and Park [23]	2009	31	3 weeks	13.3 ± 4.9 months	93.55%
Moriue et al. [24]	2008	5	-	>6 months	100%
Matsumoto et al. [25]	2010	61	9.3 months	10 months	91.8%
Erdogan [26]	2011	21	6–10 months	-	100%
Okada and Okada [15]	2012	106	≥5 days	4.6 months (range 2–12 months)	92.45%
Moon et al. [27]	2013	15	-	9 months (range 5–12 months)	86.67%
Tseng et al. [28]	2013	43	2–3 months	6 months	95.35%
Kim et al. [29]	2013	21	2–3 weeks	Foreseen: 12 weeks Later: 37.9 ± 21.3 weeks (range 16–84 weeks)	90.5% (12 weeks) 57.14% (range 16 to 84 weeks)
Park et al. [30]	2014	31	From 2–3 weeks or more (41 days)	161 days	77.4% *
Guler et al. [31]	2015	74	Until cure and correction nail curve	12.7 ± 3.9 months	91.9%
Arik et al. [32]	2016	41	4–6 weeks	Range 6–12 months (8.6 ± 2.1 months)	80.5% *
This study		46	6 months	6 months	78.26% *
**Nail reeducation techniques**					
Lloyd-Davies and Brill [1]	1963	100	-	2 years	60% *
Wallace et al. [12]	1979	Study 1 (retrospective): 25. Study 2 (prospective): 36	6 weeks10–12 weeks	6 months	52% (retrospective study) * 56% (rrospective study) *
Cameron [33]	1981	100	3.5–4 months	6 months	61% *
Senapati [34]	1986	25	-	2–56 weeks (of 23.7 ± 14.3 weeks)	79% *
Connolly and Fitzgerald [35]	1988	61	-	2.5 years	72% *
Reijnen y Goris [36]	1989	Stage I: 20 Stage II: 47 Stage III: 52 Total: 119	From 2 weeks until pain disappeared	2 years	96% (stage I and II) * 38% (stage III) *
Ilfeld [37]	1991	43	-	-	97.68% *
Salasche et al. [38]	1998	62	-	2 years	100% *
Lazar et al. [39]	1999	20	3–9 weeks	-	95% *
You et al. [40]	2001	27	-	6 months	37% *
Gupta et al. [41]	2001	39	-	6 months	79.5% *
Abby et al. [42]	2002	28	-	4 months	71.4% *
Kim et al. [43]	2003	Group 1: 28 Group 2: 29	Group 1: 3 days Group 2: 2 weeks	1 year	92.8% (treatment of 3 days) * 89.7% (treatment of 2 weeks) *
Ozawa et al. [44]	2005	9	2 weeks	6–37 months (average 17.7 months)	89.89% *
Nazari [45]	2006	32	7–15 days	6 months	93.75% *
Lee et al. [46]	2011	30	-	Average 8.42 months	60% *
Doğan et al. [47]	2013	16	2 weeks	6 months	100% *
Ceren et al. [48]	2013	57	15 days	6 months	87.8% *
AlGhamdi and Khurram [49]	2014	23	1 months	6 months	80% *
Taheri et al. [50]	2014	11	≥4 weeks	5 months	81.82% *
Gutiérrez-Mendoza et al. [51]	2015	10	2 months	2 months	80% *
This study		46	6 months	6 months	78.26% *

Note: * The results of this study and those that are close to it.

**Table 4:** Studies of spicule technique effectiveness and the nail curve correction with nail reeducation techniques.

**Spicule Technique Effectiveness**					
**Studies**	**Year**	**Nº Cases**	**Treatment Time**	**Follow-Up Time**	**Effectiveness Rate**
Maeda et al. [52]	1990	22	15.9 months (range 3–48 months)	6 months	22.73%
Stoduto and Palomo [53]	2014	32	-	-	90.62%
This study		48	6 months	6 months	8.33%
**Nail Curve Correction Studies**					
**Studies**	**Year**	**Nº Cases**	**Nail Curve Correction**	**Observations**	
Di Chiacchio et al. [9]	2006	25	3.04 mm	Index of nail width increases and index of height decreases after treatment	
Lee et al. [46]	2011	30	-	-	
Kim et al. [29]	2013	21	-	Transversal nail width decreases and width index is maintained after treatment.	
This study		46	1.8798 mm	Nail width mean decreases in the follow-up period (without orthonyxia). Tendency to increase nail curve.	
